# A Sensor Platform for Athletes’ Training Supervision: A Proof of Concept Study

**DOI:** 10.3390/s19183948

**Published:** 2019-09-12

**Authors:** Alessandro Zompanti, Anna Sabatini, Marco Santonico, Simone Grasso, Antonio Gianfelici, Bruno Donatucci, Andrea Di Castro, Giorgio Pennazza

**Affiliations:** 1 Unit of Electronics for Sensor Systems, Department of Engineering Campus Bio-Medico University of Rome, 00128 Rome, Italy; a.sabatini@unicampus.it (A.S.); m.santonico@unicampus.it (M.S.); s.grasso@unicampus.it (S.G.); g.pennazza@unicampus.it (G.P.); 2 Sport Medicine and Science Institute, CONI (Comitato Olimpico Nazionale Italiano), 00197 Rome, Italy; antonio.gianfelici@gmail.com (A.G.); dicastro.training@gmail.com (A.D.C.)

**Keywords:** athletes’ performances, low power sensor, wireless system, lactate

## Abstract

One of the basic needs of professional athletes is the real-time and non-invasive monitoring of their activities. The use of these kind of data is necessary to develop strategies for specific tailored training in order to improve performances. The sensor system presented in this work has the aim to adopt a novel approach for the monitoring of physiological parameters, and athletes’ performances, during their training. The anaerobic threshold is herein identified with the monitoring of the lactate concentration and the respiratory parameters. The data collected by the sensor are used to build a model using a supervised method (based on the partial least squares method, PLS) to predict the values of the parameters of interest. The sensor is able to measure the lactate concentration from a sample of saliva and it can estimate a respiratory parameter, such as maximal oxygen consumption, maximal carbon dioxide production and respiratory rate from a sample of exhaled breath. The main advantages of the device are the low power; the wireless communication; and the non-invasive sampling method, which allow its use in a real context of sport practice.

## 1. Introduction

A gas sensor array for volatile fingerprinting (dubbed e-nose) and e-tongue systems (based on cyclic voltammetry analysis) are used in several applications.

E-nose is applied in several fields: from the medical practice for the early detection of respiratory diseases [1], to the evaluation of food freshness [2] and the monitoring of the air quality [3]. The e-tongue systems are used for voltammetric analysis in the discrimination of the quality of olive oil [4], wine [5], water [6] and also physiological liquids [7].

Those systems have not been used, so far, for the evaluation of sport activities.

In sport activities the monitoring of the physiological parameters allows one to evaluate the physical condition of the athletes. The current state of art of this research field does not follow a unique guideline, but several approaches are pursued and different theories are reported to support the effectiveness of each parameter proposed for athletes’ monitoring [8].

The accepted and available approaches can be divided into two groups: the first one based on a self-report measurement of the physical effort (e.g., Borg Scale), and the second one that consists of the objective monitoring of certain physiological parameters. In the first approach the athlete has to score his physical effort using a standard scale; the second method is performed via specific measurement instruments. These two approaches can also be jointly applied. In the application of the second approach, the anaerobic threshold is often used. It is a well-known parameter for the assessment of sportive performances, defined by Mader as the 4 millimoles per litre concentration of lactate [9]. There is also evidence about the use of other phisiological parameters, such as heart rate [10], respiratory rate [11] and other respiratory parameters for the evaluation of the physical effort.

However, in spite of the lack of a standard and shared methodology, lactate concentration is the most used [12]. This fact is also evident by the availability of many different instruments for the measurement of lactate in the blood extracted by the ear lobe [12].

A key-point in the application of lactate evaluation is represented by the invasivity of the technique, which is based on blood analysis. Here, the ability of a sensor device to estimate the lactate concentration via the analysis of a saliva sample, which, of course, involves a non invasive collection procedure, is demonstrated. A step up in the field should be given by the simultaneuos application of non invasive and easy-to-use sensors able to implement the measurements of many other physiological parameters. Feasibility of this approach is granted by the design and development of dedicated electronic interfaces for sensors’ interactions as a collaborative sensor system, via low-power strategies and low-noise circuits for signal acquisition and treatment. The aims of this work are: (1) the demonstration of the feasibility of a method to estimate lactate concentration in humans by the analysis of saliva, with a proof of concenpt study showing the effectiveness of the saliva sampling procedure and the relevance of the instrumental output in the estimation of the lactate concentration; (2) the feasibility of a system approach based on more than one sensors in order to enlarge the system’s accuracy and effectiveness in the monitoring of athletes’ training.

## 2. Materials and Methods

During the experiment, samples of saliva and of exhaled breath were collected. Saliva was analyzed using the sensor array for liquid analysis of a sensor system named BIONOTE [13]. Exhaled breath has been measured using a sensor array for volatile analysis of the BIONOTE system. Both these systems, named BIONOTE-L and BIONOTE-V respectively, are briefly described below. Further details can be found in the references.

The BIONOTE-V used in this set-up is based on 20 MHz quartz microbalances, the module is composed of 7 QMBs (Quartz MicroBalance) covered by anthocyanins extracted from different plant tissues, such as blue orthesia, red cabbage and red rose [13]. The breath is stored in a Tenax cartridge [14], after collection performed via Pneumopipe^®^ [15] and analyzed with a desorbing process run consecutively at 50, 100, 150 and 200 °C. Between the desorbing steps, a flow of nitrogen is distributed inside the measurement chamber. The final output for each measurement is an array of 28 numbers. These 28 numbers are the frequency shifts registered by each of the 7 QMBs for the four temperatures of desorption. More technical information about the most relevant parameters of these acoustic sensors can be found in literature [13,16,17].

The BIONOTE-L performs voltammetric analysis on the saliva samples, using 3 screen-printed electrodes made of gold (working electrode), silver (reference electrode) and platinum (counter electrode): the system has a low power, about 80 mW, 3.6 V, 22 mA and a size of about 12 cm^2^. These features, the order of magnitude of the power and size, allow the use of the system in wearable applications.

**Figure 1:**
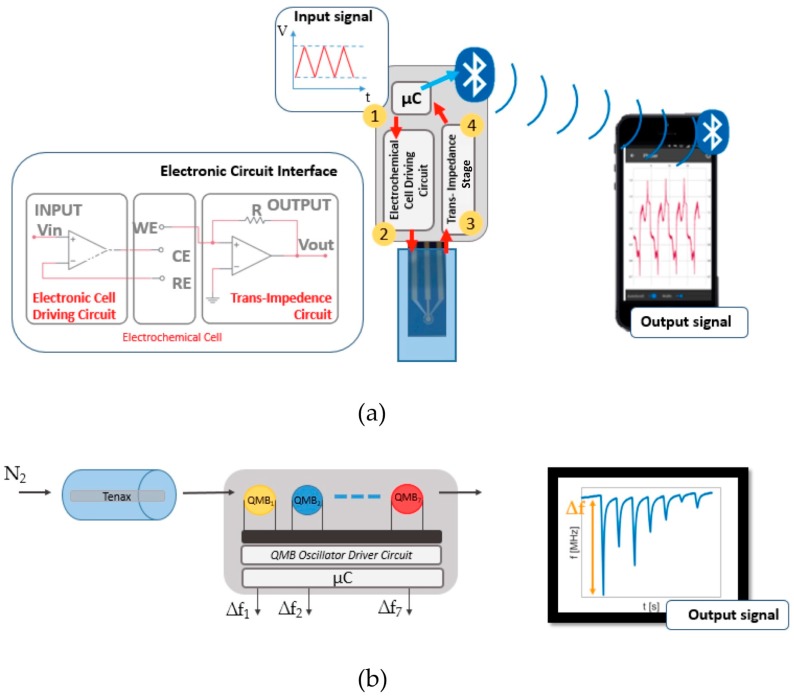
Device used in the experimental set-up: (**a**) BIONOTE-L (**b**) BIONOTE-V.

In the case of BIONOTE-L, Data, acquired with a microcontroller, are transmitted using a BLE (Bluetooth Low Energy) module to an external device, like a smartphone or tablet. The input signal is a triangular wave ranging between −1 and 1 V with a frequency of 0.01 Hz. That signal is generated by the microcontroller and the electrode is driven by a custom electronic interface. In Figure 1 the structure of the sensor used in this study is reported [18].

When the input signal is applied to the solution, a redox reaction is induced and the output signal is measured in the working electrode. A current flows from the counter electrode to the working electrode and it is converted to a voltage value using a trans-impedance stage. The output of the sensor is an array of 500 numbers.

The samples of saliva and of exhaled breath were collected during the performing of a modified Mader test. Twelve male triathlon athletes, trained in running, swimming and cycling, performed a running test on a treadmill designed for the evaluation of the athletes’ maximum effort. The study was in collaboration with the Italian National Olympic Committee (Comitato Olimpico Nazionale Italiano) Sport Medicine and Science Institute, CONI, Rome. The modified test is composed of 5 minutes running at different increasing speeds (12, 13.5, 15 and 16.5 km/h) and each step is separated from the next one by 2 minutes’ pause. During the pauses saliva samples are collected using a sampling device called Salivette^®^ [19]. The athletes were requested to gently chew the cotton roll for one minute to stimulate saliva production and then to put it in the plastic tube. The extraction process was performed with the centrifugation of the sampler for 2 minutes at 1000 RCF and room temperature. The extracted liquid volume of 0.5 mL could be frozen to allow post measurement, or directly diluted in deionized water and analyzed: standard disposable spectrometry cuvettes, with a volume of 4 mL, were used; in order to ensure that the electrodes were correctly dipped into the liquid solution, saliva samples were diluted into the minimum required volume of 3.75 mL. Before the analysis of each sample of saliva, a white sample of deionized water was measured.

**Figure 2:**
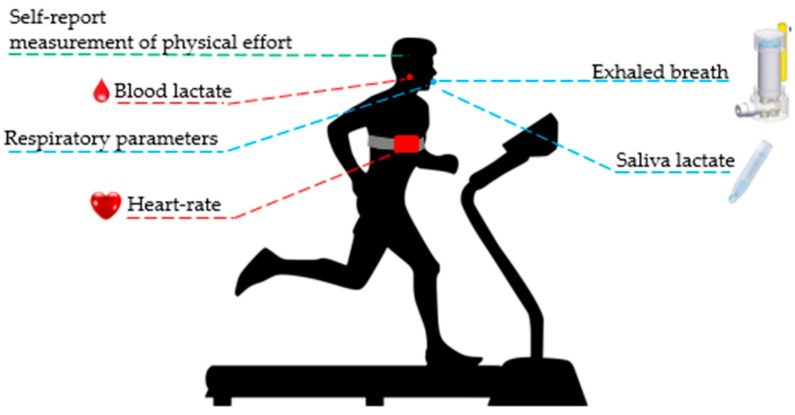
The athlete is running on a treadmill; Respiratory parameters and heart rate are real-time monitored. During the pause between the steps, lactate from blood and saliva samples are evaluated; at the beginning and at the end of the test as well. An exhaled breath sample is also collected.

The exhaled breath was collected at the beginning and at the end of the test and it required the athletes to breathe normally for 3 minutes into the Pneumopipe [16]. The cartridge was stored at 4 °C until the analysis with the BIONOTE-V (Figure 2).

Output data have been used for the elaboration of the models to predict several physiological parameters (blood lactate concentration, maximal oxygen production, maximal carbon dioxide production, respiratory ratio, etc.). Both supervised and unsupervised analyses were performed, specifically PCA (principal component analysis) and PLS-DA (partial least squares discriminant analysis), using the PLS Toolbox in the MATLAB environment. PCA and PLS-DA are two data analysis techniques normally used to treat and elaborate multidimensional data-sets. They represent an optimal solution to extract relevant information by the output of arrays of chemical sensors [20], by reducing the number of variables via a linear combination of the original parameters in the direction able to maximize information. The model calibration was performed using measurements collected during the execution of the modified Mader test. Specifically the physiological values measured are: VO_2_—oxygen uptake; VCO_2_—carbon dioxide production; Pet O_2_—oxygen partial pressure; Pet CO_2_—carbon dioxide partial pressure; FR—respiratory rate; RQ—respiratory ratio (produced CO_2_/O_2_ uptake); VT—tidal volume; Ti—duration of inspiration; and Te—duration of expiration. These parameters were real-time monitored, during the whole duration of the test, using a Quark CPET platform (COSMED srl, [21]): The Quark CPET is a state-of-the-art metabolic cart for gas exchange analysis (VO_2_, VCO_2_) either during exercise testing or resting protocols. The platform is equipped with a paramagnetic sensor for the O_2_, and an infrared sensor for the CO_2_. The athletes have to breathe into a sensor-embedded mask connected to a workstation that is able to record and evaluate data (Figure 2).

Several PLS models were built analyzing the data obtained from exhaled breath samples and saliva samples.

Two models were built using data obtained analyzing saliva samples with BIONOTE-L: the models were built using only the output data obtained from the BIONOTE-L; therefore, a 500-column matrix was used. The model was cross-validated using the leave-one-out method. The first model was built to predict lactate concentration in the whole range of variation for the parameter (from 0 to 10 mmol/L); the second model was built to measure the lactate in the range of 2-6 mmol/L. The same goals were achieved with two models built using a data-set obtained from the data fusion of data collected measuring both saliva samples (using BIONOTE-L) and exhaled breath samples (using BIONOTE-V), thus a 528 column matrix was used. A linear normalization was performed on the dataset, but no feature selection was performed. All the models were cross-validated using the leave-one-out method.

The lactate values were measured via an earlobe blood sample collected by a doctor during the pauses.

## 3. Results

Data collected from both BIONOTE-L and BIONOTE-V were analyzed using an unsupervised method, principal component analysis (PCA), in order to evaluate the method’s effectiveness. Then, a supervised analysis, the partial least squares discriminant analysis (PLS-DA), was used to build predictive models for the determination of saliva lactate levels and respiratory parameters. During the calibration process, measured blood lactate concentrations and respiratory parameters were used to build the models.

### 3.1. PCA Analysis

**Figure 3:**
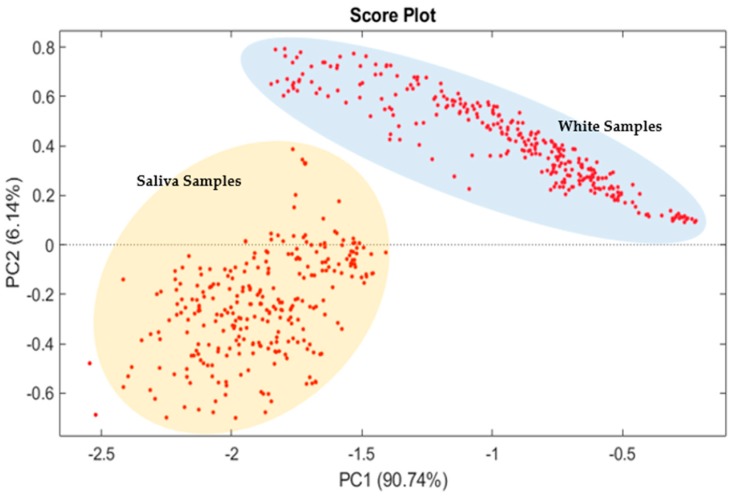
Principal component analysis (PCA) analysis of saliva and white samples are evaluated using a PCA method. Here the plot score of the first two principal component is reported. It contains almost the 97% of the explained variance. Here, two clusters can be distinguished: the blue one is composed of white samples while the orange cluster is made of saliva samples.

Data obtained from BIONOTE-L, measuring saliva samples and white samples of deionized water, were analyzed using PCA. The scope of this elaboration was to check if the procedure for saliva collection and treatment is effective: is the fingerprint of a saliva sample obtained by its measurement with the BIONOTE-L different from another (similar) standard solution? If yes, as the PCA model demonstrates, it could be possible to distinguish among different saliva samples. Figure 3 shows the results: on the plane representing the score-plot of the first two principal components of the calculated model, the information is mainly contained in PC1 (90.6% of the information); two clusters can be distinguished, one for the saliva samples and the other for the white samples.

Thus, PCA analysis shows the effectiveness of the sampling method and the ability of the BIONOTE-L to discriminate saliva samples from white samples.

### 3.2. PLS-DA Analysis

Several PLS models were built analyzing the data obtained from exhaled breath samples and saliva samples.

**Figure 4:**
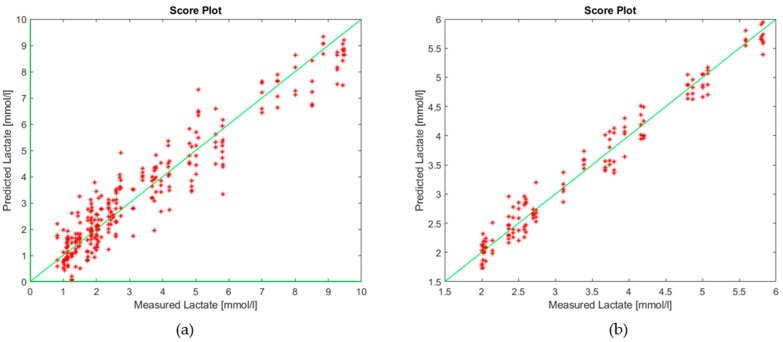
Score plot of (**a**) lactate in the range of 0–10 mmol/L; (**b**) lactate in the range of 2–6 mmol/L.

Two models were built using data obtained analyzing saliva samples with BIONOTE-L; their performances are reported in Table 1. The first model was built to predict lactate concentration in the whole range of variation of the parameter (from 0 to 10 mmol/L); it presents a RMSECV (root mean square in cross validation) error of 1.94 mmol/L. The second model was built to measure the lactate in the range of 2–6 mmol/L; this range is critical for the evaluation of the anaerobic threshold in order to improve the athletes’ performances. In this case, the RMSECV error decreases at 0.66 mmol/L. The score plots of the models are reported respectively in Figure 4a,b.

Several models were built using data obtained analyzing exhaled breath samples with BIONOTE-V; six respiratory parameters can be predicted using these models. The errors are reported in Table 2.

**Figure 5:**
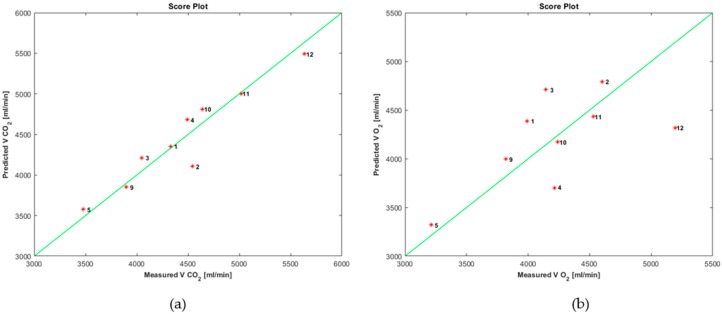
Score plot of: (**a**) VCO_2_; (**b**) VO_2_ using data from exhaled breath.

Figure 5 shows the score plot of the parameters VCO_2_ and VO_2_.

The same models were built using a data-set obtained from the data fusion of data collected measuring both saliva samples (using BIONOTE-L) and exhaled breath samples (using BIONOTE-V). The data-fusion models present RMSECV errors reported in Table 3.

## 4. Discussion

The BIONOTE can be used for the detection of the anaerobic threshold and for the monitoring the athletes’ performances. The physiological parameter can be predicted using different models developed in this work. The lactate concentration error is 1.94 mmol/L and it decreases in the range of interest, 2–6 mmol/L, at 0.66 mmol/L. This value is larger than the error claimed by other commercial devices, as shown in Table 4, but its advantage is that BIONOTE is non invasive and easy-to-use, so the method used for the acquisition of the data does not need the involvement of expert medical staff. This promising result of saliva analysis also paves the way to a fruitful application for disease diagnosis, as already demonstrated with other sensors [22]. The respiratory parameters’ models obtained with the exhaled breath data show a higher error in cross validation compared to models obtained with exhaled breath and saliva data. The device and the set-up can be improved and this could give a reduction in the error in the model. Further work could be focused on developing an experimental set-up to allow direct sampling from the mouth of the athlete, or adding a functionalized layer that can improve the detection of the lactate for a specific application. By the way, it must be considered that the current method for exhaled breath collection and desorption is mediated by an adsorbing cartridge. The contribution of the exhaled breath fingerprint given by BIONOTE-V is important for athlete monitoring, but the exhaled breath sampling and measurement procedure should be further developed in order to be integrated in a same device with the BIONOTE-L. Obviously this aspect is beyond the scope of this paper. Besides, it is a useful reminder that the same wireless BLE solution used for the BIONOTE-L has already been tested for the BIONOTE-V. The device’s size and low power consumption allows the use of the device in an IoT (Internet of Things) system in which each athlete can be monitored remotely by a trainer or a doctor which can evaluate his performance, which can also be done by comparing it with respect to other sportsmen.

It is worth a remark that for the real application of this method based on saliva and exhaled breath collection and measurement, two points have to be clarified in future experiments: (1) the confirmation of the method’s effectiveness for women as well (herein a male population has been tested); (2) testing the effectiveness of this monitoring ‘action’ for supporting an improvement in the training procedures of the athletes.

## 5. Conclusions

The BIONOTE sensor platform can be used for the detection of the anaerobic threshold and for the monitoring the athletes’ performances, measuring blood lactate concentration from saliva samples and respiratory parameters from exhaled breath samples.

Even if the device shows a measurement error higher than the error claimed by other commercial devices, it has the great advantage to be non invasive and easy-to-use: so it can be used without the needing of medical staff. The contribution of exhaled breath fingerprint is important to achieve a more inclusive monitoring of the athletes’ performances: however further developments are required to allow direct sampling of exhaled breath samples, without the mediation of adsorbing cartridges.

The device’s size and low power consumption allows the use of the sensor platform in an IoT (Internet of Things) system in which each athlete can be monitored remotely by a trainer or a doctor which can evaluate his performance, which can also be done by comparing it with respect to other sportsmen.

## Figures and Tables

**Table 1:** Model parameters obtained from saliva analysis.

	Range	Latent Variables	RMSECV
Lactate	0–10 mmol/L	7	1.94 mmol/L
Lactate	2–6 mmol/L	35	0.66 mmol/L

**Table 2:** Model parameters obtained from exhaled breath analysis.

	Range	LVs	RMSECV
VCO_2_	3000–6000 mL/min	3	720 mL/min
VO_2_	3000–5500 mL/min	2	894.55 mL/min
Pet O_2_	100–125 mmHg	3	4.71 mmHg
Pet CO_2_	30–45 mmHg	3	2.49 mmHg
ReRa	0.95–1.2 [mL/min]/[mL/min]	4	0.04 [mL/min]/[mL/min]
VT	2–3 L	3	0.66 L

**Table 3:** Model parameters obtained using a fusion of saliva and exhaled breath data.

	Range	LVs	RMSECV
VCO_2_	3000–6000 mL/min	2	1024 mL/min
VO_2_	3000–5500 mL/min	2	894 mL/min
Pet O_2_	100–125 mmHg	4	6.11 mmHg
Pet CO_2_	30–45 mmHg	3	2.46 mmHg
ReRa	0.95–1.2 [mL/min]/[mL/min]	3	0.06 [mL/min]/[mL/min]
VT	2–3 L	2	0.5 L

**Table 4:** Comparison chart of the proposed device and five commercial portable devices [12].

	Manufacturer	Method	Analysis time [s]	Accuracy [within 2–5 mmol/L] [19]	Invasiveness
BIONOTE-L	ESS Lab, UCBM, Italy	Eletrochemical sensor	100	0.66	NO
Lactate Pro2	Arkray KDK, Japan	Aperometic reagent	15	0.11	YES
Lactate Scout+	EKF Giagnostics, Germany	Enzymatic amperometric	10	0.09	YES
Nova Statsrip Xpress	Nova Biomedical, USA	Electrochemical biosensor	13	0.13	YES
Edge	Transatlenticv Science, USA	Electrochemical biosensor	45	0.14	YES
i-STAT	Abbott Laboratories, USA	Amperometric	280	0.45	YES
